# Proteomic Profiling of SupT1 Cells Reveal Modulation of Host Proteins by HIV-1 Nef Variants

**DOI:** 10.1371/journal.pone.0122994

**Published:** 2015-04-13

**Authors:** Reshu Saxena, Sudipti Gupta, Kavita Singh, Kalyan Mitra, Anil Kumar Tripathi, Raj Kamal Tripathi

**Affiliations:** 1 Toxicology division, CSIR-Central Drug Research Institute, Sector-10, Janakipuram Extension, Sitapur Road, Lucknow, India; 2 Electron Microscopy Lab, Sophisticated Analytical Instrument Facility, CSIR-Central Drug Research Institute, Sector-10, Janakipuram Extension, Sitapur Road, Lucknow, India; 3 Department of Medicine, King George’s Medical University, Chowk, Lucknow, India; Lady Davis Institute for Medical Research, CANADA

## Abstract

Nef is an accessory viral protein that promotes HIV-1 replication, facilitating alterations in cellular pathways via multiple protein-protein interactions. The advent of proteomics has expanded the focus on better identification of novel molecular pathways regulating disease progression. In this study, nef was sequenced from randomly selected patients, however, sequence variability identified did not elicited any specific mutation that could have segregated HIV-1 patients in different stages of disease progression. To explore the difference in Nef functionality based on sequence variability we used proteomics approach. Proteomic profiling was done to compare the effect of Nef variants in host cell protein expression. 2DGE in control and Nef transfected SupT1 cells demonstrated several differentially expressed proteins. Fourteen protein spots were detected with more than 1.5 fold difference. Significant down regulation was seen in six unique protein spots in the Nef treated cells. Proteins were identified as Cyclophilin A, EIF5A-1 isoform B, Rho GDI 1 isoform a, VDAC1, OTUB1 and α-enolase isoform 1 (ENO1) through LC-MS/MS. The differential expression of the 6 proteins was analyzed by Real time PCR, Western blotting and Immunofluorescence studies with two Nef variants (RP14 and RP01) in SupT1 cells. There was contrasting difference between the effect of these Nef variants upon the expression of these six proteins. Downregulation of α-enolase (ENO1), VDAC1 and OTUB1 was more significant by Nef RP01 whereas Cyclophilin A and RhoGDI were found to be more downregulated by Nef RP14. This difference in Nef variants upon host protein expression was also studied through a site directed mutant of Nef RP01 _(55AAAAAAA61)_ and the effect was found to be reversed. Deciphering the role of these proteins mediated by Nef variants will open a new avenue of research in understanding Nef mediated pathogenesis. Overall study determines modulation of cellular protein expression in T cells by HIV-1 Nef variants.

## Introduction

Nef is a 27kDa, N-terminal myristoylated accessory protein of HIV-1, involved in disease progression and pathogenesis. It is expressed in early stage of viral lifecycle and is one of the first proteins to be detected after host cell invasion. It manipulates the cellular environment by MHC class I downregulation, CD4 downregulation, and benefits the virus by increasing its propagation and activating anti-apoptotic machinery [1, 2, 3]. Nef interacts with signal transduction proteins of host cell and provides long term survival of infected T cells and induces destruction of non-infected T cells through apoptosis [4, 5]. Nef also promotes the endocytosis and downregulation of cell surface proteins, including CD4 and MHC proteins [2]. This action possibly impairs cytotoxic T cell function, and advances host immune evasion by virus thereby establishing state of infection [6]. The multifunctional protein thus helps the virus overcome host immune defenses, maintain high viral loads, contributing to disease progression. Nef promotes survival of infected cells and favours viral infection and replication through interaction with cellular proteins involved in both trafficking of cell-surface receptors and signal transduction molecules [5, 7].

Accessibility of the conserved domain of Nef controls successful interaction of proteins to the host protein. There are several mutations in the conserved region and regulates functionality of Nef as the disease progresses. The mutation in the proteolytic cleavage region does not allow Nef to come in cytoplasmic region. As a result, the protein interaction in cytoplasm is not possible [3]. Myristoylation of Nef favours its association with the cytoplasmic leaflet of cellular membranes, and is essential for nearly all of its major functions [8,9]. Thus, sequence variation will contribute to structure based functional analysis of Nef and in understanding how the Nef variants vary in their regulatory role with host proteins. Nef contributes to disease progression and different stages of infection reveal specific Nef mutations [10–12]. Several studies have analysed Nef gene in HIV-1 patient’s population. Krichhoff *et al*.[13] confirmed that deletion or gross abnormalities in Nef were rarely present but comparison of Nef alleles derived from long term non-progressor and individuals with progressive HIV-1 infection revealed that specific variations were associated with different stages of infection. Sequence heterogeneity of Nef transcripts in HIV-1 infected subjects at different stages of disease was observed [14]. These results demonstrate that various Nef activities were modulated during the course of HIV-1 infection to maintain high viral loads at different stages of disease progression. Thus, it is likely that Nef variants influence the expression of host proteins in a different way and a comparative proteomic analysis will capture such alterations in cellular proteome, regulated differently by Nef variants.

Not surprisingly, persons infected with HIV-1 strains that have deletions of the nef gene, show delayed AIDS symptoms and progression, than those infected with standard HIV strains. Nef protein enhances pathogenesis by increasing viral replication. The deleted Nef viral strain, upon infection in monkey, showed low viral load, high CD4+ Tcells and inhibited progression towards AIDS [15]. Moreover, Nef-deleted provirus could be detected in individuals with slow AIDS progression [16]. Nef may therefore be a valuable target for pharmaceutical intercession in AIDS progression. Nevertheless, by all accounts, Nef is a pleiotropic protein, capable of executing diverse intracellular functions that augments the overall pathogenicity of the virus [17] including the enhancement of virion infectivity, disruption of T cell signaling, regulation of apoptotic pathways, and perhaps most importantly, alteration of cell-surface receptor expression.

Although it is widely known that HIV-1 initiates a profound global host cell response to infection, it is difficult to trail specific expression and pathway alterations occurring at protein level. HIV-1 infection of cells lead to production of foreign viral RNAs and proteins, alterations in cell cycle, morphology, physiological stress, impaired protein synthesis and degradation, hence it is reasonable to anticipate large differences in protein expression between a normal and infected cell. Proteomic techniques are able to characterize these overall changes, which lead to the understanding of the role of viral proteins in manipulating the cell. Cofactors involved in HIV-1 infection can be identified via the molecular interactions between viral and cellular proteins. Knowledge of the interplay between cellular and viral factors involved in pathogenesis plays a key role in the understanding of disease progression and such proteins could potentially arise as an early diagnostic for a disease state, or a potential therapeutic target [18].

Many proteomic studies have been reported revealing the altered protein expression upon HIV-1 infection and its associated disorders [19–24]. Proteomic studies in T cells revealed changes in protein expression and compared the differences between uninfected and HIV-1-infected T cells. Classes of proteins and general pathways that were altered upon HIV infection were identified [23,24]. Identification of these unique proteins may uncover the changes in viral state and indicate phenotypic variability in response to infection in a given population [25]. Proteins showing up regulation and/or down regulation upon HIV-1 infection have been found to be associated with alteration in cellular pathways causing metabolic rerouting. Pathway analysis revealed that the differential expression of proteins targeted selected biological pathways. Such studies help to identify surface markers on HIV-1 infected cells by performing comparative proteomics on HIV-1 infected eukaryotic cell and control cells [26].

As proteins do not function in isolation, alterations in protein networks revealed by understanding the pathway helps unveil the host mechanisms contributory to pathogenesis. Comprehensive information of HIV-1-mediated effects on host cellular protein networks and unique protein targets for the design of therapeutics is required [27]. Nef being a key contributor to pathogenesis, is anticipated to show modulation of host proteins. Recently a comparative proteomic analysis showed Nef dependent increase of virus infectivity [28]. Another study revealed interacting partners in Nef mediated nanotube formation [29]. Scoring Nef mutations and observing difference of Nef variants over host protein modulation would be instrumental in the research process. The present study is aimed to unveil how the HIV-1 pathogenicity factor Nef and mutations occurring in it are involved in such alteration in host protein network.

## Materials and Methods

### 1. Materials and Reagents

RPMI1640 (Sigma), Antibiotic antimycotic solution (Sigma),Fetal bovine serum (FBS) Gibco BRL, Pure link RNA extraction kit (Invitrogen Life Technologies), Maxima SYBR Green/ROX qPCR Master Mix(Fermentas), RevertAid Premium first strand cDNA synthesis kit(Fermentas), IPG strips (GE Healthcare), Urea, Thiourea, CHAPS, EDTA, PMSF,dithiothreitol (DTT), Protease inhibitor cocktail, Iodoacetamide, Bradfoerd, Silver nitrate, Ponceau, BSA, poly-L lysine and paraformaldehyde were purchased from Sigma Aldrich. Passive Lysis Buffer (Promega), PVDF membrane (Millipore), Luminata forte Western HRP substrate (Millipore), anti alpha-tubulin monoclonal antibody (Invitrogen Life Technologies), DAPI (Invitrogen Life Technologies), Primary antibodies- anti-Cyclophilin A antibody (ab58144), anti-eIF5A antibody [EP527Y] (ab32407), anti-ENO1 antibody (ab85086), anti-OTUB1 antibody (ab98280), anti-RhoGDI antibody [1F2] (ab118159), and anti-VDAC1/Porin antibody—N-terminal (ab135585) were purchased from abcam, UK.

### 2. Selection of Nef variants after sequencing of *nef* from HIV-1 infected patients

Sixteen HIV-1 infected individuals from King George Medical College, Lucknow, India were included in the study for sequencing of nef gene. Two Nef variants: Nef RP14 and Nef RP01 were selected for study on basis of sequence variations (NCBI Accession No.:GQ184340 and GQ184335 respectively).

### 3. Cell culturing and transfection

SupT1 (T lymphoblastoid cell line) cells were obtained from our Institutional cell line repository. Cell culture of SupT1 cells was grown in RPMI media supplemented with 10% fetal bovine serum and antibiotics at 37°C and 5% CO_2_. For transfection, 4 million cells were seeded per sample. After 24 hours, cells were centrifuged, washed twice with incomplete media and resuspended in 400 μl incomplete media. 20 μg of respective plasmid was added in the cells and electroporation was carried out at 290 V pulse (low-voltage mode). Cells were immediately transferred to flasks for further culture. After 2 hrs complete media with 20% FBS and antibiotics was added. Cells were harvested after 48 hrs and subjected for further experiment

### 4. RNA extraction and quantitative real-time PCR

Real time PCR analysis was done in SupT1 cells (vector control vs Nef transfected) with the 6 genes keeping GAPDH as housekeeping gene. Total RNA was isolated from control and Nef treated cells using Purelink RNA mini kit (Invitrogen) following manufacturer’s protocol. DNase enzyme digestion of the isolated RNA was done to remove any DNA present in it. RNA was quantified and 10 μg of RNA was taken for DNase digestion. 1 unit of DNase (Fermentas) was added to the RNA in presence of 1xDNase buffer (Fermentas) and incubated at 37°C for 10 min and then transferred to 70°C for 10 min in order to denature the DNase. DNase digested RNA (5 μg) was reverse transcribed using two step cDNA synthesis Kit (Fermentas). Briefly, real-time PCR was performed on Roche`s 480 Real Time PCR Instrument using SYBR green qPCR mix supplied with two step qRT-PCR Kit (Invitrogen) with 25 ng cDNA and 1 ul of 10 pM of specific primer sets (Table 1) for gene to be amplified in a total volume of 20 μl reaction mix. The thermal cycling conditions comprised 2 min at 50°C, 10 min at 95°C, followed by 40 cycles at 95°C for 30 s, 57°C for 30 s, and 72°C for 30 s. All the reactions were performed in triplicate. Relative quantification of the target mRNA was done using delta delta CT method followed by normalization with the level of the internal control GAPDH mRNA level.

**Table 1:** List of primers used for amplification in q-PCR.

S. No.	Gene	Primer sequence
1.	α-enolase	FP: 5’ TACCTTCATCGCTGACCTGGTTGT 3’
		RP: 5’ TTTCTGAAGTTCCTGCCGGCAAAC 3’
2.	Ubiquitin thioesterase	FP: 5’ TGATTGAGCAGGTGGAGAAGCAGA 3’
		RP: 5’ ACCCTCGATGAAGTGCTCGAAGAA 3’
3.	Rho GDI	FP: 5’ AGCATACGTACAGGAAAGGCGTCA 3’
		RP: 5’ AGCATACGTACAGGAAAGGCGTCA 3’
4.	VDAC1	FP: 5’ AAAGGTCTGCAGGGTGTGGTAACT 3’
		RP: 5’ AGCTGGAGCTCCTGGAAGCTATTT 3’
5.	Cyclophilin A	FP: 5’ ATCAGCCTGGGCAACATAGTGAGA 3’
		RP: 5’ TTTCCTGCCTCTGCCTACCTTTGA 3’
6.	EIF5A	FP: 5’ TGGCAGATGACTTGGACTTCGAGA 3’
		RP: 5’ GCCTTTGAGCACCACAAAGCCATT 3’

### 5. Two-dimensional gel electrophoresis 2-DGE

#### Sample preparation

Transfection of Nef (RP14) was carried out in SupT1 cells. Rate of transfection was seen through expression of transfected cells after 24 hrs till 48 hrs. Sup-T1 cells with vector (pYFP-N1) transfection were kept as control. After 48 hours incubation, cells were harvested by centrifugation at 1500 rpm for 5 min at RT. Cells were washed twice with 1X PBS with centrifugation at 1500 rpm for 5 min at RT. Traces of PBS were completely removed and cells were immediately subjected to lysis for protein extraction. 10 million cells were lysed by 3 ml of Urea Lysis Buffer(7 M urea, 2 M thiourea, 4% (w/v) CHAPS, 1mM EDTA, 1mM PMSF,100 nM dithiothreitol (DTT) and protease inhibitor cocktail 10μl/ml) with updown mixing by pipetting. Cells were kept for lysis on rotor for 1 hr at RT, followed by centrifugation at 14000g for 20 min at 4°C-8°C. This was followed by ultracentrifugation at 1 lac g for 1 hr. Pellet was discarded and supernatant was subjected to acetone precipitation to further remove the impurities. 4V of pre-chilled acetone was added to the supernatant, taken out in separate tubes and incubated overnight at -20°C. This acetone mix was centrifuged at 13000g for 10 min at 4°C, supernatant was decanted and pellet further washed with 90% pre chilled acetone. After two washings pellet was air dried properly and resuspended in Rehydration Buffer (7 M urea, 2 M thiourea, 4% (w/v) CHAPS). Protein was quantified by Bradford method.

#### 2D Gel electrophoresis

Two-dimensional gel electrophoresis 2-DGE was carried out using IPG strip gels (GE Healthcare Bio-Sciences) on an IPGphor unit followed by the second dimension using hand-cast polyacrylamide gels on a sodium dodecyl sulfate-polyacrylamide gel electrophoresis (SDS-PAGE) vertical electrophoresis unit. Rehydration mix containing 75 μg of total soluble protein was prepared with rehydration buffer containing 2% DTT, 0.5% (v/v) pH 3–10 IPG buffer in a final volume of 300 μl. A trace of bromophenol blue (BPB) was added and the whole mixture kept at room temperature (RT) for 5 min followed by pipetting into a 13 cm strip holder tray. IPG strips (pH 3–10; 13 cm) were carefully placed onto the protein samples, covered with a lid, and placed into the IPGphor unit. The IPG strips were placed gel-face down onto the protein samples avoiding air bubbles, followed by overlaying with1 ml cover fluid and allowed to passively rehydrate with the protein samples overnight. Strips were then transferred on an IPGphor unit for IEF. The whole procedure was carried out at 20°C, and a total of 69562 Vh (volt hour) were used for the 13 cm strips. Following IEF, the IPG strips were removed from the strip holder and the cover fluid adsorbed on filter papers. The strips were then immediately used for the second dimension. The strip gels were incubated in equilibration buffer (6 M urea, 75 mM Tris-HCl pH 8.8, 29.3% glycerol, 2% SDS, 0.002% bromophenol blue, 200 ml) containing 2% (w/v) DTT for 15 min with gentle agitation, followed by incubation in the same equilibration buffer supplemented with 2.5% (w/v) iodoacetamide for the same time periods as above at RT. For the second dimension, the IPG strips were rinsed with running buffer (0.025 M Tris, 0.192 M glycine and 0.2% (w/v) SDS), placed onto pre-casted 12% polyacrylamide gels and overlaid with agarose solution (60 mM Tris-HCl, pH 6.8, 60 mM SDS, 0.5% (w/v) agarose, 0.01% (w/v) BPB). The gel cassettes were placed onto the PAGE running assembly. Electrophoresis was carried out at 80V for first 2 hrs and then increased to 120 V till end. To visualize the protein spots, the polyacrylamide gels were stained with silver. Staining with silver nitrate was performed as described in the instructions provided with the Plus One Silver Staining Kit (GE Healthcare).

#### Image acquisition and analysis

Protein patterns in the gels were recorded as digitalized images using a digital scanner. The stained gels were scanned wet and all images saved were cropped to include only the resolving area of the gels. The gels were quantitated in profile mode as instructed in the operating manual of the ImageMaster 2D Platinum software (GE Healthcare). All the gels were aligned against a prominent common spot and images were analysed to identify valid spots. Spots were numbered and spot density graphs were acquired through the software techniques. Three gels of treated and control group were analyzed together to find significant up regulation or down regulation of spots.

### 6. Identification of Proteins in 2D gel spots

The spots showing significant down regulation upon Nef infection were identified by LC-MS/MS and further studied through bioinformatics tools. Silver stained protein spots showing differential expression pattern were picked and sent to (Proteomics International Pvt. Ltd, Nedlands, Western Australia) for doing mass spectrometry. In brief, protein samples were trypsin digested and peptides extracted according to standard techniques [30]. Peptides were analysed by electrospray ionisation mass spectrometry using the Ultimate 3000 nano HPLC system [Dionex] coupled to a 4000 Q TRAP mass spectrometer [Applied Biosystems]. Tryptic peptides were loaded onto a C18 PepMap100, 3 mm [LC Packings] and separated with a linear gradient of water/acetonitrile/0.1% formic acid (v/v). The amino acid sequence tag obtained from each peptide fragmentation in MS/MS analyses was analysed to identify proteins of interest using Mascot online search engine (www.matrixscience.com) against the taxonomy set to Homo sapiens (human).

### 7. Western blotting

Western blotting was done in Sup-T1 cells. Transfected cells (Nef RP14 and Nef RP01) and control cells (pYFP-N1 vector) were harvested by centrifuging cells at 1500rpm for 5minute at 4°C. Cells pellet was washed twice with 1X PBS by centrifugation at 1500 rpm for 5min at 4°C. PBS was removed and pellet was resuspended in 100μl of 1X Passive Lysis Buffer (Promega) and sonicated at 30 amplitude giving 4 pulses of 5 second on and 5 second off. Cell lysate was centrifuged at 12000rpm for 20min at 4°C to remove cell debris. Supernatant was collected and protein estimation was done using Bradford method. 100μg of protein sample was mixed with 6X Laemmli sample buffer. The mixture was heated for 2 min in boiling water bath and total protein was run on 12% SDS-polyacrylamide gels in a vertical slab gel unit was assembled using the manufacturer’s instructions (Amersham Biosciences). The gel was electrophoresed initially at 10 mA till the samples reached resolving gel and thereafter at 20 mA till the end. The gel was removed from the apparatus and western blots were prepared by transferring the separated proteins from the polyacrylamide gel onto a PVDF membrane (Millipore) in a semi dry transfer carried out at 15 V for 3 hrs. Post transfer the membrane was stained with Ponceau stain to check protein transfer and rinsed twice with triple distilled water to remove the stain. Membrane was incubated in blocking buffer (5% BSA in 0.01% PBST-pH 7.4) for 2 hrs at room temperature with gentle shaking followed by incubation with appropriate dilution of the primary antibody in 1% BSA containing PBST buffer dilutions according to manufacturer's instructions) overnight at 4°C. The blots were washed 6 times with 0.05% PBST buffer, for 5 min each and were then incubated with appropriate dilution of the respective secondary antibody in PBST buffer (dilutions according to manufacturer's instructions) for 2 hours at room temperature with gentle shaking. The blot was washed 7–8 times with 0.05% PBST buffer (pH 7.4), for 10 min each. Protein was detected using chemi-luminescent substrate provided in Luminata forte substrate kit (Millipore-1 ml/membrane). Substrate was added to the membrane kept for 1 min, extra solution was drained out, exposed to UV-light and image was captured. Blots were then stripped and probed with an anti alpha—tubulin monoclonal antibody (Invitrogen) as control for equal loading of proteins.

### 8. Immunofluorescence Study

Immunoflurescence studies were conducted in SupT1 cells. Control and Nef transfected cells were harvested, resuspended in media and overlaid on 0.01% poly-L lysine coated coverslips placed in 12 well cell culture plates. Cells were allowed to adhere on these coverslips for 6 hrs at 37°C. Media was removed and cells were washed thrice with 1X DPBS. Cells were fixed by adding 4% paraformaldehyde for 20 min at 37°C, washed and were then blocked by 5% BSA in 1X PBS for 1 hr to remove non-specific binding sites. Primary antibodies at recommended concentrations in PBS were added over the cells and incubated overnight at 4°C with gentle shaking. Cells were washed and respective Secondary fluorescent antibodies were added at 1:500 dilution. Plate was covered with foil to maintain dark and kept for shaking for 2 hrs at RT. Washing was done and coverslips were mounted with DAPI on a slide and sealed with nail paint. Slides were incubated in dark for 1 hr at RT and Immunofluorescence was detected by Zeiss Confocal microscope using 65X objective lens with 4X and 2X zoom and analysed by Zeiss LSM Image viewer software.

### 9. Nef RP01 mutant generation with mutation at 55–61 aa

Nef RP01 mutant was constructed by replacement of 55CAWLEAQ61 amino acids to 55 AAAAAAA61. For mutant generation nef gene was amplified using two primer sets as shown in Table 2. First set of primers amplified the N- terminal fragment of Nef, while second primer set amplified the C- terminal fragment of Nef. These two PCR amplified products with mutated overlapping regions, were mixed and used as template for amplification of final mutated nef gene with primers: Nef RP01 FP and Nef RP01 RP. The amplified mutated nef gene product was cloned in pYFP-N1 for further experiments. Desired mutation was confirmed by DNA sequencing.

**Table 2:** 

S.No.	Primer	Primer Sequence
**1**	**Primer Set 1**	
	Nef RP01 FP	5’ATCAAGCTTATGGGGGGCAAGTGGTCAAAAAGC3’
	Nef RP01 mut1 RP	5' TGCTGCCGCCGCGGCAGCATCAGCATTAGTT 3'
**2**	**Primer Set 2**	
	Nef RP01 mut2 FP	5' GCCGCGGCGGCAGCAGCAGAG 3'
	Nef RP01 RP	5’ACTGTCGACACGCAGTCTTTGTAATACTCCGGATGT3’

### 10. Bioinformatic analysis

The sequence alignment of protein translates of Nef variants was done online by Clustal Omega software (http://www.ebi.ac.uk/Tools/msa/clustalo/). Sequences were uploaded in FASTA format as Protein Sequences and analysed further for variability. The function of the identified underexpressed proteins was elucidated by SWISS-PROT database and the interaction between these proteins and Nef was checked by HIV interaction database http://www.ncbi.nlm.nih.gov/RefSeq/HIVInteractions. A protein-protein interaction network of the 6 proteins with closest interacting partners was generated through STRING 9.1.(string-db.org) Partners with high confidence level and score were interconnected.

### 11. Statistical Analysis

Numerical data was tested for statistical significance using paired student’s t test (two-tailed). Differences were considered significant when p ≤ 0.05(*), and very significant when p ≤ 0.01(**). All data was analyzed by graphpad-prism 5.

### 12. Ethics Statement

The Ethical clearance for the study was granted by Human Ethical Committee of Research Cell of King George Medical University, Lucknow, India (Ref Code: *XVIII ECM/P9*). The written informed consent was obtained from all study participants and this consent procedure was approved by the Ethics Committee.

## Results

### 1. Analysis of sequence variability in HIV-1 Nef variants

**Fig 1:**
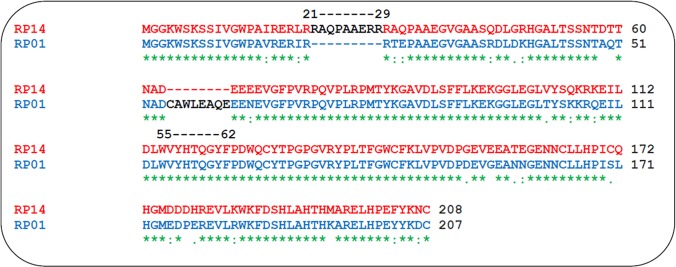
Sequence alignment of the HIV-1 Nef (RP14) with Nef (RP01): RP14 and RP01 protein translate sequences are shown in red and blue colour respectively. The blue colored 21–30 are the nine amino acids insertion in RP14, and 55–61 of RP01 is the position where seven amino acids deletion in RP14. Amino acids found common are starred while amino acids with difference are shown with dot upon alignment.

The preliminary objective of the analysis was to identify sequence variability, and major variations in Nef sequences to understand the differential functions of Nef in HIV-1 patients. The two Nef variants taken in this study were RP14 and RP01. Besides other single point variability, RP14 exhibit a nine amino acid insertion at 21–29 aa position and a significant deletion of 7 aa at 55–61 position. Interestingly, this deletion involves the CD4 downregulation and proteolytic cleavage site ^55^CAWLEAQ^61^ that is known to be well conserved in Nef. Hence, RP14 was considered mutant form, while RP01 with sequence close to wild type Nef was taken as wild type Nef. The sequence alignment showing the variability between these variants is shown in Fig 1. This unique mutation has not been correlated to disease progression in the literature so far and could not determine the stage of HIV-1 pathogenesis so its implications have to be investigated. The two Nef variants cloned in pYFP-N1 displayed full biological activity as shown in our previous reports, and were thus functional [31–33]. These were studied further through proteomics approach.

### 2. Difference in host protein expression mediated by Nef as revealed by 2DGE

**Fig 2:**
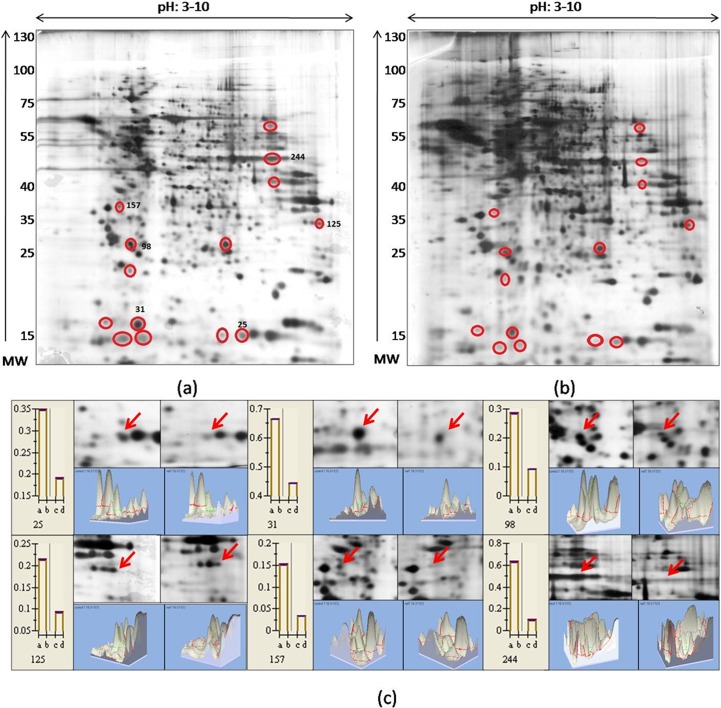
Proteome profile of SupT1 cells after Nef Transfection. 2D gel electrophoresis image of (a) vector (pYFP-N1) transfected SUPT1 cells vs (b) Nef RP14 transfected cells. The pI scale was constructed based on the dimensions of the linear pH gradient strip (pH range 3–10 linear over 13 cm).Red circles indicate spots showing differential expression. Spots showing major downregulation in Nef transfected cells have been marked as spot no. 25, 31, 98, 125, 157 & 244. (c) Panel shows enlarged section of spots and their relative spot density and 3D peak area. Results are expressed as mean 6 SEM, n = 3. The difference in protein spot density between treated and control group was assessed by Student’s t-test. The pH increases from left to right and the molecular mass decreases from the top to the bottom of the gels.

2D gel electrophoresis offers a suitable method to understand the modulation of host proteins as mediated by Nef, through quantification of the differences in protein expression of control and Nef transfected cells. To evaluate the host cellular factors being affected by Nef, proteome analysis of Nef transfected SupT1 cells was carried out through 2D gel electrophoresis. Nef RP14 transfected cells and pYFP-N1 transfected vector control cells were harvested after 48 hours of transfection, whole cell protein lysates of each were subjected to 2D gel electrophoresis. The gels with spots are shown in Fig 2(A) & 2(B). Gels were scanned and analysed through Image Master 2D Platinum software (GE Healthcare). 2D gel platinum software Spot a corresponding to β-actin (PI 5.29 and MW 41.7 KDA) was considered as one of the marker protein, selected for aligning the gels and normalization of loading. Experiment was performed in triplicate. On image acquisition, total 516 spots were identified in 2D gel of cells, of which, 375 spots were found to be common in both the gels. 14 unique spots were found to show differential protein expression upon Nef transfection. Density of the spots was calculated through software and comparison of spot densities revealed that Nef significantly altered the expression of host proteins. These proteins were found to be downregulated upon Nef transfection. 6 spots (spot no. 25, 31, 98, 125,157 & 244) showing major downregulation were picked and sent for LC/MS-MS. Other spots could not reach significant statistical value due to variation in spot densities among three different experiments and were thus not considered for further analysis. Enlarged sections of the region of gel showing altered expression and the degree of difference in spot density and peak area of 3D images are displayed in Fig 2(C).

### 3. Identification of differentially expressed proteins

Differentially expressed proteins of interest were excised and successfully identified through LC-MS/MS followed by mascot software analysis. The proteins were considered identified on basis of high sequence coverage and score based on peptide matching. The spots with more than one protein hit identified through software, protein hit with highest score and sequence coverage were considered as protein identified. The proteins were identified as Cyclophilin A, (Peptidyl-prolyl cis trans isomerase), Eukaryotic translation initiation factor 5A-1 isoform B, rho GDP-dissociation inhibitor 1 isoform a, voltage-dependent anion-selective channel protein 1, Ubiquitin thioesterase protein OTUB1,and α-enolase isoform 1(ENO1). The molecular weight and PI of each protein was determined. As shown in Table 3 and S1 Fig, all the 6 proteins identified had 3 or more peptides matched upon spectrum analysis and showed high sequence coverage. The matched peptides are shown bold red. According to annotations from Uniprot (Swiss-prot/EMBL) and descriptions provided, identified proteins were found involved in various cellular functions like host-virus interaction, Protein and mRNA transport, apoptosis, signal transduction, ion transport, protease activity, GTPase activation, immune response, plasminogen activation and energy associated pathways like glycolysis. The detailed description of the identified proteins is shown in Table 3.

**Table 3:** Protein Identification Summary.

Spot No.	Protein Name	Protein Symbol	Molecular^a^ mass(KDa)	pI^b^	Peptides^c^ count	Sequence^d^ Coverage (%)	Protein score ^e^	Fold change^f^ (downregulation)	Biological/ Molecular function^g^	Cellular location^h^
**25**	Cyclophilin A(Peptidyl-prolyl cis trans isomerase)	CYPA (PPIA)	17.99	7.68	5	23	91	1.82	Host-virus interaction/ peptide binding, peptidyl-prolyl cis-trans isomerase activity	Cytoplasm
**31**	Eukaryotic translation initiation factor 5A-1 isoform B	EIF5A-1	16.82	5.08	3	16	83	1.5	Protein biosynthesis, Protein transport, Translocation mRNA transport/Elongation factor	Cytoplasm. Nucleus. Endoplasmic reticulum membrane; Peripheral membrane protein
**98**	rho GDP-dissociation inhibitor 1 isoform a	ARHGDIA (RhoGDI)	23.19	5.02	3	17	89	3.11	Rho protein signal transduction, anti-apoptosis, cellular component movement/ GTPase activation	Cytoplasm
**125**	voltage-dependent anion-selective channel protein 1	VDAC1	30.75	8.62	6	21	203	2.35	Apoptosis, Host-virus interaction Ion transport/ Porin	Mitochondrion outer membrane. Cell membrane
**157**	Ubiquitin thioesterase protein	OTUB1	35.15	5.4	4	16	178	5.03	Adaptive immunity, Ubl conjugation pathway/Hydrolase, Protease	Cytoplasm
**244**	α-enolase isoform 1	ENO1	47.13	7.01	10	29	413	6.32	Glycolysis, Plasminogen activation Transcription, Transcription regulation/Lyase, Repressor	Cytoplasm. Cell membrane

List of proteins downregulated by Nef in 2D gels shown in Fig 2(A) and 2(B)

a & b Theoretical mass and isoelectric point calculated by Swissprot database at http://ca.expasy.org/sprot/

c Number of peptides detected by mass spectrometry for each identified protein

d Sequence coverage (%) means the number of amino acids spanned by the assigned peptides divided by the sequence length.

e Mascot score for identified protein

f Compared with control SupT1 cells (vector transfected)

g Protein function from SWISS-PROT database

h Protein location from SWISS-PROT database

### 4. Real Time PCR for analysis of transcriptional profile of the differentially expressed proteins

**Fig 3:**
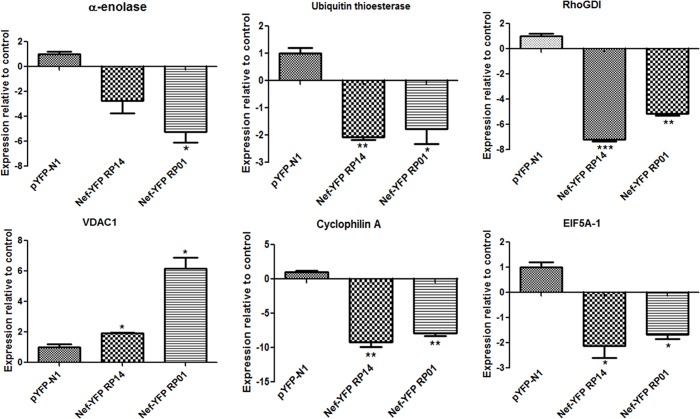
Nef induced transcriptional alteration of the six genes measured by qPCR. Real Time RT-PCR was performed to analyse the expression of the six genes. Fold change in expression upon Nef transfection is presented in respect to control. Except VDAC1 all 5 genes were downregulated by Nef variants. The values and error bars represent average and standard deviations of three independent set of experiments. Student T test was performed to find out significant difference between control and treated conditions.

Quantitative Real Time PCR was done in order to check the transcriptional alterations of the 6 genes, keeping GAPDH as control housekeeping gene. Significant down regulation of 5 genes was obtained in Nef treated SupT1 cells as compared to control and were consistent with the result of 2DGE. In contrast, VDAC1, showed different result and was found to be upregulated at transcript level. As shown in Fig 3, both Nef variants caused significant downregulation of rest of 5 genes with Nef RP14 causing greater than Nef RP01 in case of CYPA, EIF5A, RhoGDI and OTUB1, however the downregulation of ENO1 was greater by Nef RP01. Fold change in mRNA expression of the genes was normalized with GAPDH and calculated from three different experiments.

### 5. Validation of the identified proteins by western blotting analysis

**Fig 4:**
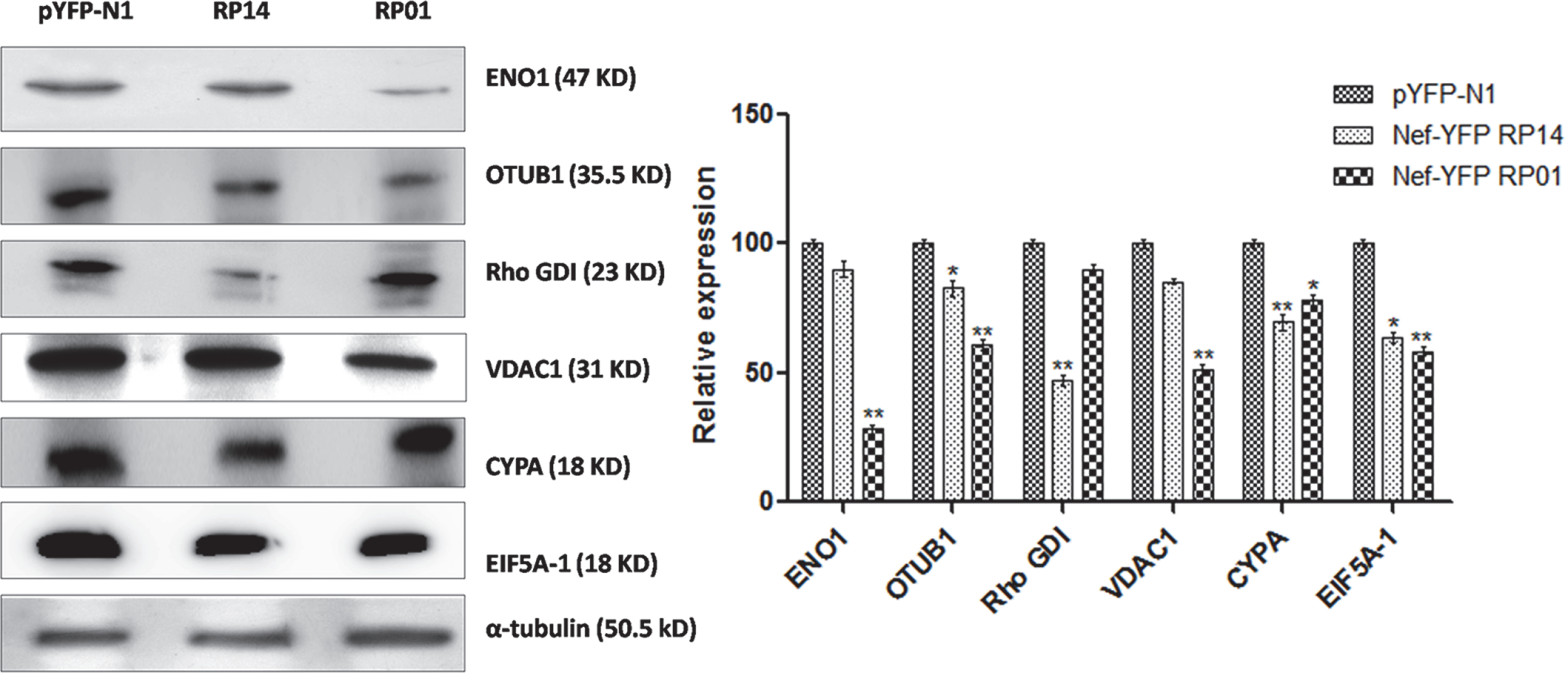
Western blot confirmation of LC-MS/MS identified proteins. Western blotting of representative proteins revealed similar quantitative profiles as suggested by 2DGE analysis. Contrasting effect of Nef variants observed upon expression of the six proteins- Cyclophilin A, EIF5A-1, Rho GDI, VDAC1, OTUB1, and ENO1. alpha tubulin was used as a loading control. Data are presented as the mean ± SD of three independent experiment.

To further validate the proteomics result and ensure that identified proteins were underexpressed by Nef, western blotting was performed with specific antibodies against the proteins of interest in control and Nef transfected SupT1 cells. All the 6 proteins showed downregulation by Nef variants. Interestingly, there were striking differences in the effect of the Nef variants upon the expression of these proteins. As demonstrated from Fig 4, α-enolase (ENO1), VDAC1, and ubiquitin thioesterase (OTUB1) were more underexpressed by Nef RP01 with fold decrease 3.55, 1.95, and 1.63 respectively (p<0.01); whereas the downregulation of Cyclophilin A and RhoGDI was greater by Nef RP14 with fold decrease 1.43 and 2.12 respectively (p<0.01), as calculated from three different blots normalized with alpha tubulin as loading control. Underexpression of EIF5A-1 was found to be similar by both Nef forms. Thus, western blotting results confirmed the 2DGE results and showed difference of Nef variants upon expression of these proteins.

### 6. Confirmation of proteomics result and comparison of the surface expression of proteins through immunofluorescence study

**Fig 5:**
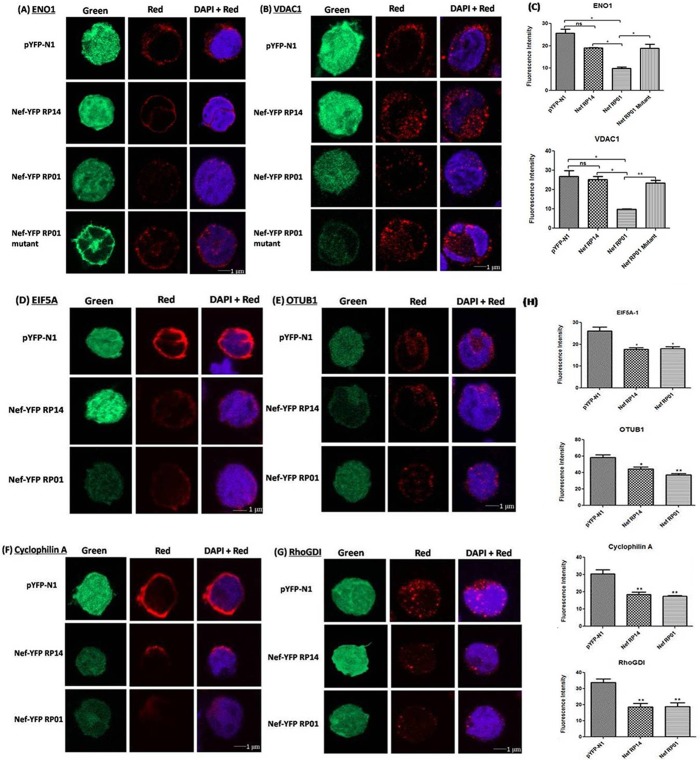
Immunofluorescence staining of SupT1 cells showing comparative expression of downregulated proteins. Immunofluorescence study to compare cell surface expression of six proteins in SupT1 cells (1 μm section) was captured by Confocal microscopy at 40 X and 63 X magnification. Figure shows immuostained SupT1 cells for expression of Vector/Nef (green), respective proteins (red) with their nucleus stained with DAPI (blue). Fluorescence intensity was measured for 5–8 Nef/vector transfected cells from 6 different fields of each sample and mean was calculated. Data is presented as values from two different experiments. Fig 5 displays ENO1 and VDAC1 (A-C) and EIF5A, OTUB1, CYPA and RhoGDI (D-H).

Control and Nef transfected cells were fixed, permeabilised and labelled with specific antibodies against the six proteins followed by incubation with fluorescent secondary antibodies and fluorescence was captured through confocal microscopy to compare the expression of the 6 proteins in SupT1 cells being transfected with Nef variants. As seen in Fig 5 the results of immunofluorescence were found to be consistent with proteomics. On comparison of fluorescence intensity of the labelled protein over cells there was significant downregulation seen in the protein expression by Nef. α-enolase (ENO1), VDAC1 and OTUB1 were underexpressed substantially by Nef RP01, whereas EIF5A1, Cyclophilin Aand RhoGDI were underexpressed by both Nef variants. Cells with vector/Nef expression (Green channel) specific protein expression (Red Channel) and DAPI stained nucleus are shown in Fig 5 with statistical significance of fold decrease being calculated from fluorescence of 5–8 transfected cells taken in 6 different fields of which single cell image has been shown in figures.

### 7. Mutation of 55CAWLEAQ61 in RP01 reversed its downmodulating effect on α-enolase (ENO1) and VDAC1

**Fig 6:**
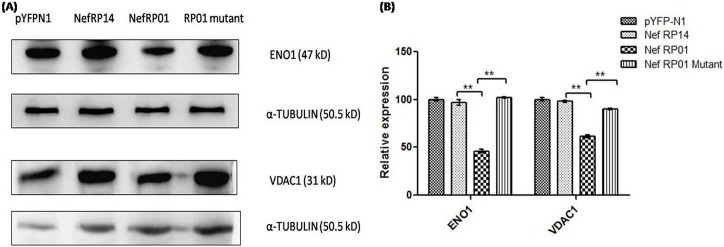
Western blotting analysis with Nef RP01 (55AAAAAAA61) mutant. (A)Western blots showing reverse effect of Nef RP01 mutant upon downmodulation of α-enolase (ENO1) and VDAC1 as caused by Nef RP01. Alpha-tubulin was used as a loading control. (B)Graphs representing the difference in effect of Nef RP14 Nef RP01 and Nef RP01 mutant upon expression of α-enolase (ENO1) and VDAC1. Data are presented as the mean ± SD of two independent experiment.

To investigate whether the differences observed in effect of two Nef variants over expression of the proteins, could be attributed to the unique deletion of proteolytic cleavage site in RP14, a mutant of Nef RP01 was constructed in which the amino acids of 55CAWLEAQ61 domain of Nef RP01 were replaced with Alanine. Western Blotting and immunofluorescence studies were performed to show the effect of this RP01 mutant upon expression of α-enolase (ENO1) and VDAC1 as both these proteins were significantly downregulated by RP01 but not by RP14. As shown in Fig 6, RP01 downregulates α-enolase (ENO1) and VDAC1 but RP01 mutant does not and its effect is comparable to that of RP14. This indicate the significance of 55CAWLEAQ61 site of RP01 upon these protein expression, as mutation of this site in RP01 nullified its downmodulating effect on α-enolase (ENO1) and VDAC1. Similarly, Nef RP01 mutant was employed for IF studies in α–enolase (ENO1) and VDAC1, and showed reverse effect on their expression as compared to RP01 (Fig 5).

### 8. Bioinformatic analysis and protein interaction pathway for Nef variants with host proteins

**Fig 7:**
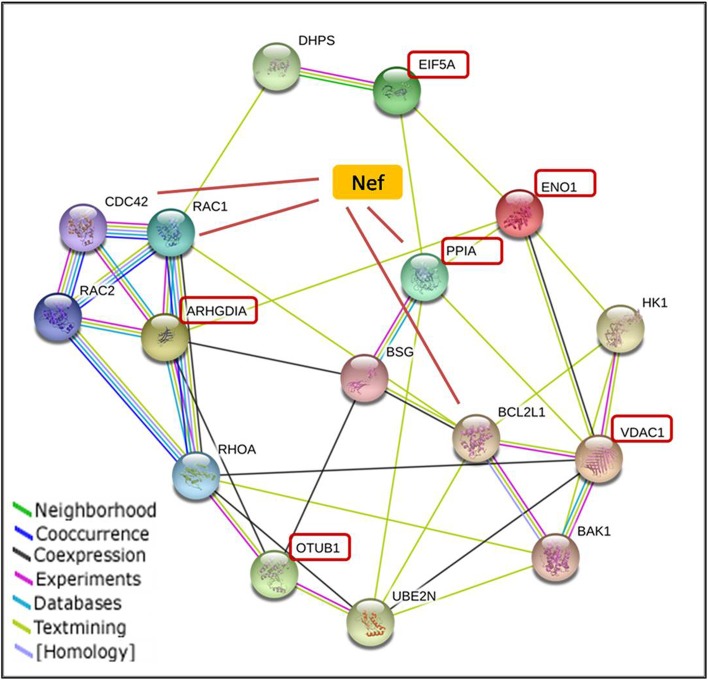
Protein-protein interactions of identified Nef downregulated proteins. Predicted network map for Cyclophilin A, EIF5A-1, Rho GDI, VDAC1, OTUB1, and ENO1 with their top partners and association with HIV-1 Nef was created in STRING. Proteins in boxes were underexpressed proteins identified in our work. Rest of proteins were host proteins from the database. Protein labeled in Yellow is Nef, with known interaction with Cyclophilin A, RAC1, CDC42 and BCL2L1 (connected with red line) as given in HIV-1, Human Protein Interaction Database.

The six proteins were found to play critical roles in regulation of different cellular functions. The Nef variants showed difference in effect over expression of six proteins. The downregulated proteins and the protein interacting partners connected in a pathway were determined by STRING 9.1 database shown in Fig 7. Direct interaction of the 6 proteins and their close interacting partners in the network generated by STRING was checked by HIV human protein interaction database and were found to be associated with Nef. Among the six proteins being downregulated, Cyclophilin A was found linked with Nef, whereas from the close interacting partners BCL2L1, CDC42 and RAC1 were associated with Nef. As demonstrated from this network Nef might be affecting the associated pathways and Nef variants must be regulating different signal transduction pathways and hence affecting viral pathogenicity differently. Further mechanism behind the regulation of these proteins by Nef and their downstream pathways, which may favour viral replication, needs to be explored extensively. Possibility of a common signalling event mediating the downregulation at transcriptional level was pursued through in silico studies of promoter sequences of the 6 genes. Sequence alignment showed 37–58% similarity among the promoters (data not shown) but no common transcription factors or significant sequence similarity was seen through this analysis.

## Discussion

HIV-1 Nef, a key determinant contributing to viral pathogenesis, binds to a diverse group of host cell signalling molecules and regulates the surface expression of numerous T-cell molecules involved in HIV-1 infection and T-cell functions [34–37]. Several conserved motifs of Nef mediate association with cellular factors and modulates protein expression through interaction with cellular kinases and signalling molecules [2]. Nef is known to induce a state of activation in the host T-cell, the principal target of HIV-1 infection, which leads to enhanced viral replication.

The present study uses a proteomics approach to investigate pathways associated with Nef regulated host proteins and understand how the sequence variability in Nef, altered its ability to modulate the protein expression in T-cells. For the study, Nef was sequenced from randomly selected HIV-1 infected patients but do not elicit any specific mutations, that could correlate to specific stage of infection. However, a conserved domain was found to be deleted in many HIV-1 infected patients. The Nef protein having deletion of conserved domain was pathogenic [31] and higher order tetramer association was found [32]. Interestingly insertion of 9 amino acids in this mutant form of Nef, RP14, was found in a variable region of patient’s nef gene. The deletions and insertions being present in N-terminal unstructured region conserves the overall length of Nef and explains that the length of N-terminal region could be critical for its functionality and restoration of Nef activities[38].

This unique deletion of 7 amino acid ^55^CAWLEAQ^61^ in unstructured region of patients nef gene compromises two functions of Nef, that is, CD4 down regulation [39] and liberation of membrane Nef to cytosolic Nef [40], which is demonstrated by mutational studies in this region [39,41]. Till date, in-vitro studies showed that Nef was cleaved by viral proteases at cleavage site located between 57W/L58, and determine the modular organization of Nef, separating it into anchor and core domain [41]. This indicates that in HIV-1 infected condition, membrane bound Nef is liberated to cytosolic Nef by cleaving at this site [42]. The studies have shown that the anchor domain N-myristoylation region of Nef, places it to cell and associates with rafts where several signalling molecules present interact for activation of T-cells [43]. The two variants have been investigated for functional aspects of Nef in our previous reports [31–33]. One study identified the interacting domain of Nef (RP01) and ASK1 where their physical interaction led to inhibition of ASK1 enzymatic activity [33]. Nef RP14 has been reported to show tetramer association [32] and showed pathogenesis in *C*. *elegans* [31] reported by our group.

Two Nef variants, Nef RP14 and Nef RP01, were selected for further study with an aim to explore how mutations occurring in Nef contribute to its functionality. Comparative proteomic study was carried out focused on the target proteins of these Nef variants and analysed the modulation of host protein expression in Sup-T1 cells to observe the difference in functional aspect of these Nef variants. Proteomic profiling allows formation of new hypothesis regarding the functionality of Nef. It is conceivable that, besides known interactions of Nef, there may be some proteins that are differentially regulated by Nef and have undescribed effects over viral life cycle.

To study the T-cell–Nef interaction, SupT1 cells cultured in-vitro were used, which likely differ from primary T-cells found in-vivo, but were effective at representing the principal effects of Nef. Proteomic profiling of SupT1 cells upon Nef transfection was done by 2D gel electrophoresis with Nef RP14, the mutant form. Differentially expressed proteins were observed and host proteins downregulated by Nef were picked and identified for further study. Six proteins with major downregulation were identified as α-enolase isoform 1 (ENO1), ubiquitin thioesterase, cyclophilin A, voltage-dependent anion-selective channel protein 1, Eukaryotic translation initiation factor 5A-1 isoform B and rho GDP-dissociation inhibitor 1 isoform a. The underexpression of these proteins by Nef needed to be further validated, and also the transcriptional alteration of these genes was to be checked to observe the level of modulation. Further confirmation involved both Nef variants, to elucidate their difference over host protein expression. Q-PCR studies were carried out to check whether this alteration was occurring at transcript level or a post-transcriptional change. All genes were found to be downregulated by both Nef forms, except VDAC1 which was found to be enhanced at transcript level.In some cases change in mRNA levels and protein levels did not correlated that well mainly due to the regulation control at different stages. Increased mRNA level parallel with downregulation of protein expression is generally seen among proteins being downregulated by ubiquitination [44]. Western blotting analysis of the six proteins by both variants showed interesting result and gave striking difference in effect of both variants over the expression of these proteins. This contrasting effect of Nef variants was further confirmed by immunofluorescence studies. This also compared the cell surface expression of the proteins. Downregulation of α-enolase (ENO1), VDAC1 and OTUB1 was more significant by Nef RP01 whereas, Cyclophilin A EIF5A-1 and RhoGDI were found to be downregulated by both Nef variants more by Nef RP14. Consistent results were obtained in Western and IF analyses for all the proteins.

The sequence variability in Nef variants RP14 and RP01 could be targeted to predict the possible mechanism behind the differential regulation of host proteins. To answer the reason behind observed difference in effect of Nef forms upon expression of host protein, especially in α-enolase (ENO1) and VDAC1, the 55–61 amino acid site representing proteolytic cleavage domain of Nef was exploited. RP14 shows natural deletion of this site apart from over 20 other point mutations as compared to RP01. In our later part of study we constructed 55CAWLEAQ61 to 55AAAAAAAA61 mutant of RP01 and checked its effect upon protein expression. Further studies showed that effect of RP01 got reversed significantly upon protein expression after generation of this mutation. It is possible that small differences between protein expression profiles reflect a high degree of specificity in mode of activation and influence cellular outcome. The proteins were found to be involved in several cellular pathways and associated with relevant host functions. The proteome of Nef infected T-cells was changed particularly in networks associated with energy release, transport, apoptosis, cell migration and signal transduction.

Most significantly underexpressed protein α-enolase (ENO1) is known to induce cell migration and tissue invasion [45–47]. In a previous report, α-enolase (ENO1) was found to be associated with HIV-1 revealed by proteomic analysis of HIV-1 virions produced by monocyte derived macrophages [21]. In present study, it was found to be downregulated by Nef, which may alter the migratory property of immune cells and affect their invasiveness. Another protein VDAC-1 (voltage dependent anion selective channel protein 1) plays role in ion transport and regulates apoptosis. This protein is believed to form the major pathway for movement of adenine nucleotides through the outer membrane and to be the mitochondrial binding site for hexokinase and glycerol kinase. It is proapoptotic energy associated regulatory protein [48–50]. Nef downregulates it and alters the energy requirement of cells that favour establishment of infection stage. Both VDAC1 and α-enolase (ENO1) are energy associated proteins. Several reports prove that cell metabolism is being affected by HIV. In a study, effect of HIV on glucose metabolism was measured in human intestinal epithelial cells [51]. Glycolytic dysregulation was detected in neuronal cells upon treatment with HIV gp120 protein [52]. These studies depicted disturbed glycolytic and oxidative activities. Decreased glycolysis should induce the pentose phosphate pathway which can also be used for the synthesis of new viral particles and stress response [21]. Decreased α-enolase (ENO1) would lead to decreased T-cell migration. Inhibition of T-cell migration would protect the infected cells from host immunological attack and help in immune evasion [53–57]. The decreased energy state of infected T-cell leading to inhibition of their migration goes in agreement with the observed downregulation of α-enolase (ENO1) and VDAC1. VDAC1 also regulates cell volume and apoptosis. In fact, apoptotic proteins have been observed differentially expressed upon infection. BCL-2 appears to regulate cell death by blocking the voltage-dependent anion channel (VDAC) by binding to it and preventing the release of the caspase activator, cytochrome c, from the mitochondrial membrane. The Bcl-X(S) isoform promotes apoptosis [58]. Many proteins employ ubiquitination and subsequent proteolysis for cell cycle regulation and apoptosis [59]. OTUB1, another Nef downregulated protein, is a hydrolase playing regulatory role in preventing protein degradation by removal of conjugated ubiquitin, prevent proteolytic degradation. This isoform has been found to regulate T-cell anergy, in which T-cells were rendered unresponsive to antigen rechallenge. OTUB1 was found to be overexpressed in viral infected cells treated with cyclosporine A, showing its possible role in causing the inhibitory effect [60,61]. Decreased OTUB1 would lead to increase of protein degradation in host and regulates various cellular events and host-virus interactions. For the first time, α-enolase (ENO1), VDAC1 and OTUB1 have been reported to be modulated by HIV-1 Nef through our study.

Known HIV-1 host-virus interactions were also observed. Cyclophilin A has known interaction with Nef and is important for viral packaging and entry [62]. Cyclophiln A is known to bind human HIV-1 gag protein and is required for the HIV-1 infectivity probably by causing uncoating of the virion coat. Viruses lacking cyclophilin A completely fail to attach to target cells and are mandatory for viral adsorption on host. It is essential for viral attachment to host cells and thus for the infectivity of virus [63,64]. Preventing CypA packaging, either by the addition of CsA to particle producer cells or by the introduction of mutations in the binding region of CA, inhibits virus infectivity, demonstrating a strict requirement for CypA in HIV-1 replication. It was found to be downregulated by Nef in T cells, inferring the prevention of superinfection by virus. Another protein eIF-5A, the eukaryotic translation initiation factor is an mRNA-binding protein involved in translation elongation and is also described as a cellular cofactor of HIV-1 Rev protein, essential for mRNA export of retroviral transcripts and HIV-1 replication [65]. Rev is essential for virus replication and is required for the cytoplasmic expression of unspliced and singly spliced viral mRNAs encoding the viral structural proteins. It was found to be downregulated upon viral infection. It plays vital role in pathways involved in stress response and cell cycle progression maintaining cell wall integrity [66,67]. Study also found Rho GDI 1 (Rho GDP dissociation inhibitor) member of the family of small GTP binding proteins down-regulated. It regulates GDP/GTP exchange reactions of Rho proteins by inhibiting the dissociation of GDP and causing subsequent binding of GTP to them [68]. Thus, its down-regulation may promote viral replication through constant activation of GTPases, as they are known to be necessary for HIV-1 replication [69–71]. Rho-GDI complex expression was also found to be down-regulated in Jurkat-Tat cells and had role in signal transduction and communication [72]. Rho GDI, which has been involved in actin cytoskeleton organization, was found to be downregulated upon HIV-1 infection. The predicted protein-protein network generated by STRING, shows connection of these underexpressed host proteins with proteins related to signal transduction receptors, glycolysis, apoptosis, GTP binding proteins, cell adhesion molecules, tumour progression, cell cycle, cell differentiation and cell migration. Although some of these proteins had known interactions with HIV-1 but their Nef mediated alteration and probable association with Nef is attributed through our study.

The network showed association of Nef with host proteins and their close interacting partners. Cyclophilin A, BCL2L1, CDC42 and RAC1 were found to be directly linked with Nef. Nef shows interaction with cyclophilin A and RAC1. In a previous study it has been reported that Cyclophilin A binds to HIV-1 Nef using a biochemical assay, however the biological significance of this interaction is currently unknown[73]. Nef binds with DOCK2-ELMO1 complex to interact with RAC1 and inhibits lymphocyte chemotaxis. RAC1 inhibits Nef mediated internalization of CD80 and CD86. Active RAC1 levels are increased by Nef mediated activation of Vav [74–76]. HIV-1 Nef upregulates active CDC42 levels in dendritic cells. CDC42 and RAC1 are required for activation of Nef associated kinase and HIV-1 replication [77]. Nef enhances apoptosis in lymphocytes by downregulation of anti-apoptotic proteins Bcl-2 and Bcl-X [78,79].

How all these changes fit in with energy state of cells, migration, apoptosis, cell cycle arrest, viral production, and/or transport remains to be established and how the observed changes correlate to pathogenicity for the infected individual is also unclear.

This first 2DGE analysis of changes in the T-cell proteome upon Nef infection and comparative proteomic study of Nef variants, not only uncovered the difference in effect of the Nef variants over host protein expression but also allowed to identify many proteins potentially involved in the virus-host interactions. The presence of such host proteins being modulated by Nef variants is often the most accurate reflection of cellular response to an infection and can be correlated to disease progression. The mutational study proved the importance of proteolytic cleavage domain among other mutations between the two Nef variants and strengthened its role in affecting the functionality of the Nef variants through differential expression of host proteins. Moreover, cell culture studies are more potent at signifying the primary effects of an infection.

HIV-1 infection leads to profound changes in the cellular transcriptome and proteome. Here, the study focused on quantifying perturbations caused by Nef and its mutations and gaining insight into the molecular mechanisms behind this modulation of proteins, in further studies.

## Conclusion

In the present study, Nef-induced modulation of T-cells was characterized in terms of the resulting alterations in cell proteome. The study adopted a gel based proteomic approach to probe changed proteins that allowed for identification of physiologically relevant targets of signal transduction pathway. In this way, many of the proteins modulated by Nef, provide indications for uncovering potential virulence mechanisms. The subversion of the T-cell by Nef and alteration of cellular factors is likely to represent an important mechanism facilitating increased virion production. We have identified cellular proteins downregulated by Nef and subsequently, on basis of contrasting effects of Nef variants upon protein expression we found a specific domain of Nef (55CAWLEAQE61) regulating this differential expression. The difference in effect of Nef variants over protein expression reflect the probable differences in these Nef forms in terms of their implications over cellular functions. Nef, by downmodulation of these proteins must be compromising the associated host functions and create an environment favourable for viral propagation. The sequence variations leading to decreased Nef pathogenicity could be further explored for identifying factors regulating the protein modulation, which would help in better understanding of Nef functionality and design inhibitors. The proteins could be explored for their effect over viral infectivity and in deciphering how Nef is affecting HIV-1 infection through underexpression of these host factors. These altered proteins can serve as future diagnostic and/or therapeutic markers and be studied as potential drug targets with relevance to different stages of disease progression. As a reference to the functionality of Nef, its large scale protein expression profile may aid a full understanding of the contribution of this accessory gene in pathogenesis and could be used in the development of inhibitors of Nef action. Thus, this study can be useful in revealing the regulatory aspect between HIV-1 Nef and its variants with host cell.

## Supporting Information

S1 Fig Sequence of the Nef downregulated six proteins. Peptides which matched with the protein sequence after sequencing of spot by LC-MS/MS are indicated by red colour.
(TIF)
