# Supplementary figures and images for: Proteomic Profiling of SupT1 Cells Reveal Modulation of Host Proteins by HIV-1 Nef Variants
Source: PLoS One. 2015 Apr 13;10(4):e0122994. doi: 10.1371/journal.pone.0122994 (PMC4395413; doi:10.1371/journal.pone.0122994)

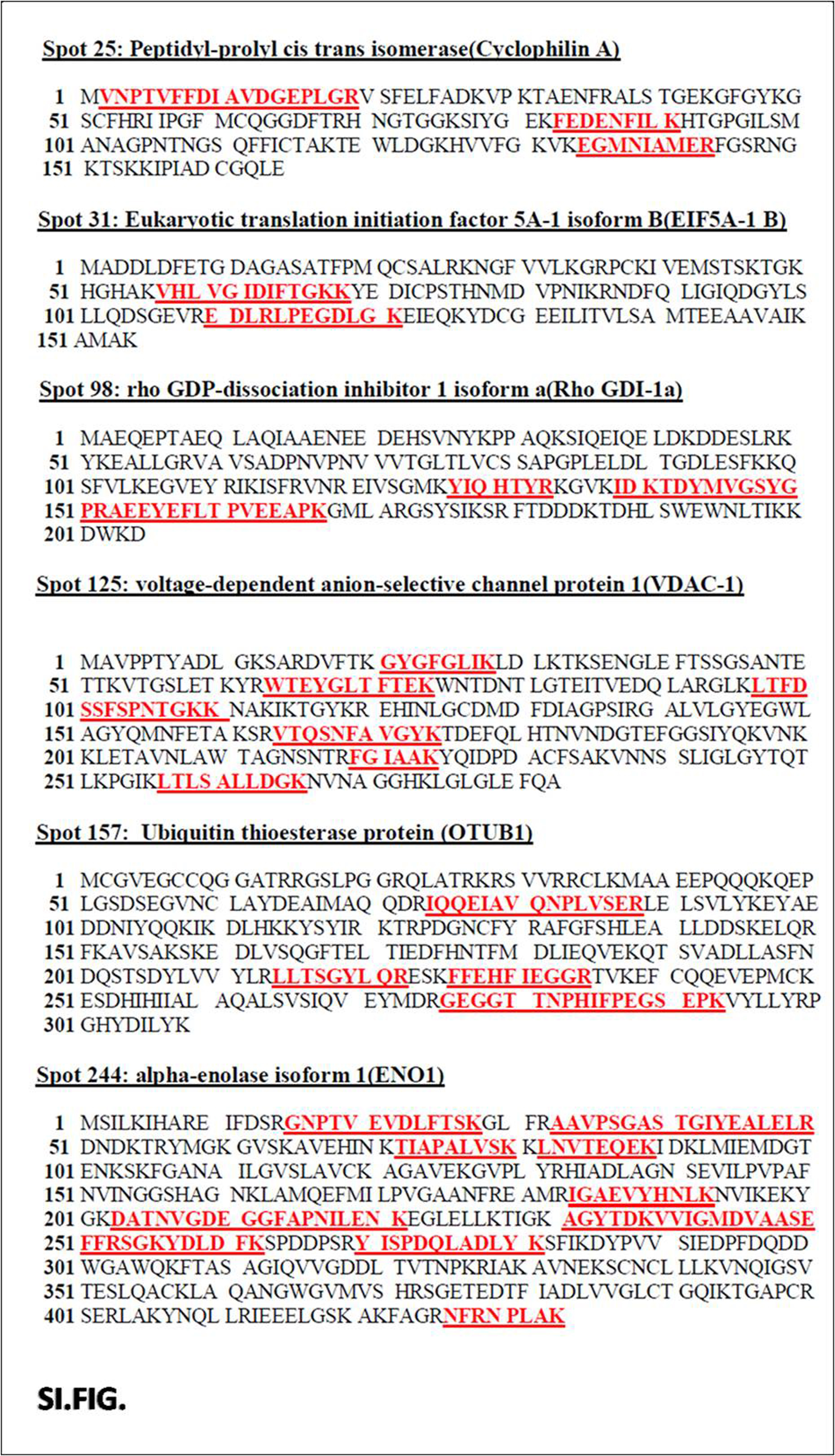

Supplement: S1 Fig — Peptides which matched with the protein sequence after sequencing of spot by LC-MS/MS are indicated by red colour. (TIF) [file pone.0122994.s001.tif]
